# Policy Series: Congressional Update

**DOI:** 10.1093/geroni/igab046.1355

**Published:** 2021-12-17

**Authors:** Brian Lindberg

## Abstract

This popular annual session will provide cutting-edge information on what the 117th Congress has and has not accomplished to date, and what may be left for end of the First Session. Speakers will discuss key issues such as pandemic relief, Social Security, Medicare, Medicaid, and the Older Americans Act.

